# Daily experiences affect the timing rather than the structure of sleep

**DOI:** 10.1093/abm/kaag003

**Published:** 2026-02-19

**Authors:** Péter Przemyslaw Ujma, Róbert Bódizs

**Affiliations:** Institute of Behavioural Sciences, Semmelweis University, Nagyvarad ter 4., Budapest, H-1089, Hungary; Institute of Behavioural Sciences, Semmelweis University, Nagyvarad ter 4., Budapest, H-1089, Hungary

**Keywords:** diary, mobile EEG, sleep homeostasis, multiday observational study, daily experiences, circadian rhythms

## Abstract

**Background:**

Sleep characteristics may be affected by daytime experiences, a fact that can be leveraged by nonpharmacological interventions to improve sleep.

**Purpose:**

To investigate the effect of daily experiences on sleep in an ecologically valid within-participant design.

**Method:**

We leverage the Budapest Sleep, Traits and Experiences Study, a large multiday observational study (*N* = 1901 nights in total) with extensive daily diaries and mobile electroencephalogram (EEG) recordings conducted for at least 7 days per participant to investigate how naturally occurring daily experiences such as social activity, emotional involvement, or mental strain affect sleep during the subsequent night.

**Results:**

The strongest influence was on the timing of sleep onset: Even after controlling for day of the week, sleep onset occurred later after more experience-rich days and pleasurable activities (*B* = 0.05-0.89 hours, *P*_max_ = .002). After statistically accounting for this extended wakefulness, we found limited evidence that daily experiences influence sleep characteristics. Only 4 effects survived correction for multiple comparisons: Sleep (*B* = 23 minutes, *P* = .002) and N3 duration (*B* = 6 minutes, *P* = .003) were longer after days with time at the workplace, rapid eye movement (REM) latency was increased after social activity (*B* = 8.6 minutes, *P* < .001), and sleep onset latency was reduced after alcohol consumption (*B* = −1.1 minute, *P* = .004).

**Conclusions:**

Our work shows that, aside from homeostatic effects resulting from extended wakefulness, sleep is relatively resilient to and only affected by a few distinct daytime experiences. Nonpharmacological interventions seeking to change sleep may need to utilize behavioral modifications exceeding normal day-to-day variation.

## Introduction

Sleep problems are common in industrialized nations, causing a significant burden on patients, health care providers, and the economy.^1^ Pharmacological means of improving sleep are ­available, but have less than perfect utility.^2–5^ Since most sleep problems are not formally diagnosed,^6^^,^^7^ these treatments are also unavailable for the majority of those affected.

Nonpharmacological methods for improving sleep are often preferable due to fewer side-effects, no risk of tolerance or dependence, the possibility of addressing root causes of sleep problems instead of symptomatic treatment, and comprehensive health benefits given that habits that improve sleep are likely to have a more general health-promoting effect.^3^ Nonpharmacological methods are also more accessible in the absence of a formal diagnosis,^8^ which is the typical case for people with sleep complaints.

Common nonpharmacological interventions include stimulus control, sleep restriction, education about sleep and good sleep habits, improving sleep hygiene, relaxation techniques, exercise, and addressing unhelpful beliefs about sleep in cognitive therapy.^8–12^ These techniques are used either in isolation or in some combination, for instance, as cognitive behavioral therapy for insomnia,^13^ low-intensity psychological interventions^14^ or sleep restriction therapy.

Nonpharmacological methods for improving sleep are based on models of sleep regulation that propose that sleep quality and structure are affected by the duration of prevailing wakefulness and the experiences the subjects had during that period. Specifically, in the 2-process model proposed by Borbély^15^^,^^16^ sleep timing, depth and duration are a function of the phase of the circadian rhythm and sleep pressure, the latter of which builds up during wakefulness in a phenomenon called process S (in contrast to process C reflecting circadian processes). The synaptic homeostasis hypothesis proposed by Tononi and Cirelli^17^^,^^18^ assigns a molecular mechanism to process S, suggesting that an increase in synaptic strength is an inevitable consequence of wakefulness and needs to be periodically downregulated during deep sleep episodes. Both the 2-process model and the synaptic homeostasis hypothesis suggest that because sleep is homeostatically regulated, increasing the duration of wakefulness is a powerful mechanism for promoting sleep.^19^ The synaptic homeostasis hypothesis opens the possibility for the experience-dependency of sleep regulation, hypothetically related to the unequal increase in synaptic strength or potentiation as a function of experiences over wakefulness periods of equal durations.

Under the latter theory of sleep regulation, it is plausible that enhancing wakefulness without changing its duration creates additional sleep pressure that improves subsequent sleep. Such effects, if well-supported scientifically, could be the basis of simple nonpharmacological interventions alleviating the disease burden caused by sleep complaints in the population. Some evidence exists about increased sleep propensity following behaviorally active days,^20^ social conflict and stress,^21^ cognitive strain,^22^ and sexual activity.^23^

Although studies of the effect of daily experiences on sleep can inform the design of nonpharmacological interventions, the ­literature is not conclusive. Most studies are limited in statistical power, the mapping of daily experiences is unsystematic and heterogeneous, and an interventional design is used, which—despite its merits in establishing causality—is limited in achievable statistical power and ecological validity.^24^^,^^25^ We have instead argued for the use of multiday observational studies in the study of daytime experiences on sleep^26^ in which the natural day-to-day within-person covariation of daily experiences and sleep is investigated.

While some multiday observational studies about the effect of daily experiences are available (e.g. Refs. ^27^ and ^28^), these generally rely on self-reports of sleep rather than objective measurements (see Refs.^29–31^ for some exceptions). In the current paper, we use the Budapest Sleep, Experiences and Traits Study (BSETS), a large, ecologically valid multiday observational study, to investigate the effects of a large array of self-reported daily experiences on subsequent objectively and subjectively measured sleep.

## Methods

### Participants

We used data from the BSETS. The full BSETS protocol has been published separately.^25^ In brief, BSETS recruited healthy volunteers to monitor their days and nights for a full week while performing their daily routine as normal. Participants were recruited using branched diffusive convenience sampling, mostly from students of Semmelweis University and relatives. As maximal ecological validity was a key goal in designing BSETS, inclusion criteria were minimal and consisted only of being fluent in Hungarian and nonrecruitment from clinical settings, in order to ensure maximal ecological validity. No intervention was given and participants were encouraged to proceed with the normal course of their lives without any restrictions on waking or sleep behavior. The Institutional Review Board (IRB) of Semmelweis University, as well as the Hungarian Medical Council (under 7040-7/2021/EÜIG “Vonások és napi események hatása az alvási EEG-re” [The effect of traits and daily activities and experiences on the sleep EEG]), approved BSETS as compliant with the latest revision of the Declaration of Helsinki. All participants gave written informed consent on a form reviewed and approved by the IRB.

### Data collection

Each evening, participants filled out a questionnaire in which they indicated (yes/no) if a series of daily experiences occurred, and subjectively rated their day (on a Likert scale) based on certain characteristics. The evening questionnaire also included a free-form diary of the events of the day. Each night, sleep was recorded with a Dreem2 mobile EEG headband. Each morning, participants filled out another questionnaire about their sleep, including subjectively rated sleep quality using the Groningen Sleep Quality Scale (GSQS).^32^ Data collection was performed between May 2021 and April 2023.

### Sleep characteristics

Each night, participants slept with a Dreem2 mobile EEG ­headband, which recorded quantitative EEG using dry silicone electrodes with a sampling frequency of 250 Hz.^25^^,^^33^ A validated^34^ proprietary algorithm scored these signals to create a hypnogram, from which we extracted the objective sleep characteristics sleep efficiency (SE), total sleep time (TST), sleep onset latency (SOL), REM latency, wake after sleep onset (WASO), the duration and percentage of N1, N2, N3 and REM sleep, as well as the number of awakenings. These encompass the most commonly used metrics of sleep macrostructure. The clock time at sleep onset (expressed as fractional hours relative to midnight) was also recorded.

In quantitative EEG analyses, we used the channel F7-O1 to calculate power spectral density in the delta (0.5-4 Hz) and low ­sigma (10-13 Hz) frequency bands using the periodogram() MATLAB EEGLAB function with 2-second nonoverlapping epochs and Hamming windows. The delta range was intended to capture slow oscillations, highly synchronized activity in deep sleep indicating sleep pressure, while the sigma range was intended to capture sleep spindles, thalamocortical oscillations in non-REM (NREM) sleep with a putative cognitive function. These were to track slow wave and sleep spindle activity, respectively. We chose this channel, as in previous studies,^19^^,^^24^ based on preliminary analyses^25^ showing a favorable tradeoff between data quality and availability. The low, as opposed to the high, sleep spindle range was chosen because it is better mapped with the frontal channel positions available with the Dreem2 device.^25^

In addition to sleep macrostructure and quantitative EEG data, we also used scores on the GSQS as an indicator of subjective sleep quality. The GSQS consists of 15 yes/no items indicating problems with recent sleep, with higher scores indicating worse sleep quality. In total, we investigated effects on 18 sleep characteristics.

### Daily experiences

In the evening diary, normally filled out immediately before sleep, participants were prompted to indicate with a Yes/No answer if any of the following 10 events happened to them (phrased in the first person singular [“I was…” or “I did…] in the original prompt):

1. They spent time in the company of others (at least 30 minutes, excluding people they normally cohabit with)
2. They were in school/university
3. They were at a workplace
4. They consumed alcohol (no data on quantity was collected)
5. They watched movies or TV series for over an hour
6. They spent at least 1 hour in front of a computer
7. They had sexual contact with someone
8. They drove a car or another vehicle for at least 1 hour
9. They spent at least 1 hour travelling by public transportation
10. They had a serious conflict or (verbal) fight with someone
  Besides these binary-coded variables, participants also noted how much time they spent outside (including walking and transportation except closed vehicles) and how much time they spent reading or playing on cell phones or other smart devices. In total 12 daily experiences were investigated.

We excluded binary-coded smart device use as this was better captured by self-reported times.

### Day ratings

In addition to specific experiences, participants marked down on a 10-level Likert scale whether their day was “Physically exhausting,” “Mentally exhausting,” “Interesting and eventful,” or “Happy” (as opposed to “Sad”). Participants were required to subjectively rate where their day could be placed along this continuum of subjective values. We used these daily ratings as further predictors of sleep characteristics, for a total of 16 experiences.

We excluded specific daily emotions, rated using the Positive and Negative Affect Schedule,^35^ as these will be part of a separate investigation.

### Statistical analysis

Although our procedures are not pre-registered, statistical analysis followed a protocol identical to previous BSETS papers.^19^^,^^24^ Analyses were hypothesis-free, using all combinations of daily experiences and sleep metrics, with correction for multiple testing.

We performed multilevel models implemented with the MATLAB fitlme() function to estimate the effects of daily experiences on sleep characteristics. A separate model for each sleep experience and each sleep characteristic was run (272 models in total). Each model estimated level 1 (within-individual) and level 2 (between-individual) effects simultaneously. Within-individual effects indicate that within the same person, sleep was different after a daily experience. Due to the time-lagged, within-participant nature of these associations they are consistent with a causal interpretation, with limitations.^36^ Specifically, the estimates are not immune to confounding: For example, it is possible that deeper sleep after a certain experience reflects not the direct effect of that experience, but its causes or its consequences. However, the time lag ensures the correct identification of the direction of (direct or indirect) causality, the within-participant design excludes confounding via time-invariant factors (eg, age, sex, or psychological traits) and the design is able to falsify causal claims.^37^ Between-individual effects, in turn, indicate that the frequency of daily experiences is associated with average sleep characteristics. These effects indicate correlations between typical sleep and daily experiences and do not imply causality.

All models were corrected for day of the week (weekend/weekday), age, sex, and lagged outcomes (the sleep metric used as the dependent variable from the previous night). Because of the well-known effect of process S on sleep characteristics, established also in BSETS,^19^ in order to separate the direct effects of daily experiences from indirect effects via extending wakefulness we also controlled all models for the duration of previous wakefulness. This was estimated by calculating the time between the last sleep epoch of the previous day’s EEG recording and the current day’s first sleep epoch.^19^ This correction was not applied for models with sleep timing as the dependent variable as late sleep timing is a cause, not a consequence, of wakefulness duration.

For a simpler analysis, we deviated from previous protocol^24^ by using a transformation instead of winsorizing and generalized (link function-based) models for the skewed variables SOL, WASO, and SE. For the first 2 variables, a log10 transformation was used. For SE, we used reflecting and log10 transforming so that the transformed value of SE was equal to SE_t_ = log10(100 − SE).

Due to the large number of models tested and the need to balance power and replicability, we employed a 2-step procedure of significance testing. We report all findings which pass a relatively lax (given the number of models) significance threshold of *P* < .01. However, we also performed a formal correction for multiple comparisons by performing the Benjamini-Hochberg correction for false discovery rate^38^ across all sleep metrics for each daily ­experience. We focus our discussion on findings on those that pass this more conservative threshold.

It is possible that the effects of daily experiences on sleep are idiographic,^39^ that is, they vary from person to person, possibly as a function of measurable characteristics. For example, it is possible that the effect of evening social interaction on sleep onset time is greater in those with an early chronotype: For these people, a late-night gathering is a major zeitgeber which substantially extends their wakefulness, while those with a late chronotype go to bed late anyway. In order to identify such idiographic effects, we re-fitted each model with significant within-participants effects using random slopes in addition to random intercepts. In such a model, not only the means (intercepts) are allowed to vary across participants but also the relationship (slope) between predictors and outcomes.^40^ Using random slopes is analogous to interactions in the sense that the effect of the predictor on the outcome is allowed to vary as a function of a third variable (here, participant ID). Significantly better fit in a random-slopes model indicates that idiographic effects are present. If random slopes provided significantly better fit (likelihood ratio test *P* < .01) than random intercepts, we calculated the correlation between individual slope estimates and all trait variables recorded in BSETS.^41^ (See the BSETS protocol paper for a complete list of trait variables.^25^) We used a significance threshold of *P* < .01 for these correlations. Slope-trait correlations show that the effect of daily experiences on sleep vary as a function of measured traits.

## Results

### Descriptive statistics

In total 267 participants took part in BSETS, of which hypnogram data were available from 258 (due to missing EEG recordings from the rest) and quantitative EEG data from 249 (due to the lack of sufficient data quality in 9). Some missingness in the data was present, see Table 1 for precise variable-wise sample sizes. Demographic information is available in Table 1. Based on scores of the Athens Insomnia Scale,^42^ a scale consisting of 8 Likert-type items with higher scores indicating worse habitual sleep quality, sleep complaints were present but in the clinical range only for a minority of participants (mean score = 4.7 points, SD = 3.4 points, with 25.9% of participants scoring more than the recommended cutoff score of 6 points). While this proportion is relatively high, similar rates have been reported in other nonclinical samples.^6^^,^^7^ Observation-level descriptive statistics, including detailed sample sizes are available in Table 1. For all analyses, we used all cases with available data for the relevant variables.

**Table 1:** Descriptive statistics for the variables in the study.^a^

		Valid *N*	Mean/%	Median	SD
**Demographics**	**Age (years)**	253	28.86	22	12.73
	**Sex (% male)**	260	45%		
	**Students (% student)**	157	58.8%		
	**Marital status—Single**	132	51.4%		
	**Marital status—Partnered**	63	24.5%		
	**Marital status—Married**	55	21.4%		
	**Marital status—Divorced**	6	2.3%		
	**Marital status—Widowed**	1	0.4%		

		Valid *N*	Mean	Median	SD	ICC	*R* ^2^ weekend	*R* ^2^ day	Day with highest value
**Sleep metrics**	**Total sleep time (min)**	1718	396.77	402.75	91.21	0.24	−0.01	1.83	Friday
	**Sleep onset latency (min)**	1718	14.84	10.50	14.70	0.31	0.01	0.10	Wednesday
	**REM latency (min)**	1712	79.38	70.50	36.79	0.23	0.23	0.16	Wednesday
	**Sleep onset time (hours)**	1718	0.44	0.18	1.79	0.49	0.39	1.71	Saturday
	**WASO (minutes)**	1718	21.01	15.00	21.57	0.38	−0.02	−0.07	Tuesday
	**Sleep efficiency (%)**	1718	91.07	92.82	6.70	0.37	−0.01	0.10	Thursday
	**NREM1 %**	1718	6.07	5.58	2.49	0.53	−0.05	0.07	Tuesday
	**NREM2 %**	1718	46.30	46.40	9.17	0.46	−0.03	−0.27	Sunday
	**NREM3 %**	1718	21.80	21.08	9.49	0.48	0.06	0.63	Wednesday
	**REM %**	1718	25.83	25.44	7.48	0.30	−0.02	0.99	Friday
	**NREM1 duration (minutes)**	1718	24.02	22.00	10.91	0.53	−0.01	0.59	Friday
	**NREM2 duration (min)**	1718	186.20	185.50	60.75	0.35	0.03	0.69	Friday
	**NREM3 duration (minutes)**	1718	83.02	84.00	31.90	0.58	0.02	−0.23	Thursday
	**REM duration (minutes)**	1718	103.53	101.50	38.49	0.25	0.01	2.80	Friday
	**Awakenings (count)**	1718	19.56	18.00	9.47	0.51	−0.03	0.44	Friday
	**Self-rated sleep (raw score)**	1745	4.13	4.00	3.41	0.20	0.27	2.05	Wednesday
	**Delta power (Log unit)**	1448	1.12	1.11	0.19	0.56	−0.01	−0.25	Wednesday
	**Sigma power (Log unit)**	1448	−0.16	−0.16	0.14	0.72	−0.07	−0.33	Saturday
**Daily experiences (continuous)**	**Time with smart devices (minutes)**	1404	72.12	45.00	89.39	0.61	0.01	−0.20	Saturday
	**Time outside (minutes)**	1743	75.58	50.00	87.49	0.39	0.00	−0.16	Saturday
	**Physically exhausting day (Likert)**	1791	4.45	4.00	2.44	0.28	−0.06	0.31	Thursday
	**Mentally exhausting day (Likert)**	1789	5.71	6.00	2.38	0.29	6.55	7.59	Thursday
	**Interesting day (Likert)**	1789	5.86	6.00	2.44	0.25	0.06	1.24	Friday
	**Happy day (Likert)**	1787	6.38	7.00	2.16	0.27	0.90	0.94	Saturday
	**Wakefulness duration (hours)**	1415	17.07	16.88	1.96	0.11	2.09	4.35	Thursday

			% days “Yes”	ICC	*R* ^2^ weekend	*R* ^2^ day	Day with highest value
**Daily experiences (binary)**	**Social interaction**	1790	80.50	0.39	4.04	5.18	Tuesday
	**School**	1788	28.97	0.56	18.16	22.26	Thursday
	**Work**	1790	24.47	0.65	8.10	8.75	Thursday
	**Alcohol consumption**	1791	18.26	0.43	0.30	3.03	Saturday
	**Watching films**	1787	39.96	0.51	1.82	2.88	Saturday
	**Computer use**	1793	63.86	0.58	3.25	4.87	Wednesday
	**Sexual activity**	1771	12.65	0.50	0.79	2.59	Friday
	**Driving**	1794	17.28	0.66	0.00	0.74	Friday
	**Public transportation**	1795	36.55	0.53	6.96	8.56	Wednesday
	**Conflict**	1789	8.11	0.43	0.65	1.43	Friday

a Medians and SDs are omitted for binary variables and the proportion of positive responses (or a highlighted reference category) is reported instead of means. ICC is calculated as adjusted *R*^2^ from either a linear regression (continuous variables) or logistic regression (binary variables, Nagelkerke *R*^2^) using participant ID as the single categorical predictor and the target variable as the dependent variable. $R_{\text{weekend}}^{2}$ and $R_{\text{day}}^{2}$ originate from similar models using binary weekday/weekend or the specific day of the week, respectively, as the single categorical predictor. $R_{\text{weekend}}^{2}$ and $R_{\text{day}}^{2}$ are expressed in percentages, not proportions, to enhance readability. Sleep onset time is expressed as hours relative to midnight. Day ratings (10-unit Likert scale) and self-rated sleep (GSQS) are expressed as raw scores. EEG power is expressed as log units. The last column shows the day of the week on which the highest values (continuous variables) or the most common observations (binary variable) were observed. For sleep variables, these indicate the previous day. For “Self-rated sleep,” the highest value indicates the worst self-rated sleep due to the coding scheme of the GSQS. REM: Rapid Eye Movement; NREM: non-REM; WASO: wake after sleep onset.

Daily experiences tended to show low to intermediate within-participant homogeneity, the lowest for “interesting” day ratings (ICC = 0.25), and the highest for driving (pseudo-ICC = 0.66) and time with smart devices (pseudo-ICC = 0.61). We created a correlation matrix of all sleep metrics including wakefulness duration, binary-coded daily experiences, and day ratings (Supplementary data, item “Zero-order correlations”). This table reports within-participant correlations in the lower and between-participant correlations in the upper triangle, and ICCs in the diagonal. Pearson correlations are reported for pairs of continuous variables, point-biserial correlations for continuous-binary variable pairs, and tetrachoric correlations for pairs of binary variables.

The co-occurrence of binary-coded experiences (based on tetrachoric correlations) and the point-biserial correlations between binary-coded experiences and day ratings were low to moderate, but in the expected direction. For example, days with car driving tended to preclude public transportation (*r* = −0.481), and days with time in the company of others were rated as more interesting (*r* = 0.248), happier (*r* = 0.138), and more physically exhausting (*r* = 0.121). Days with work were rated as more mentally exhausting (*r* = 0.274), while days with conflicts were rated as less happy (*r* = −0.108). Days with movie watching, computer use, and more time with smart devices had negative-signed correlations with other daily experiences and daily ratings, except for movie watching being slightly positively correlated with sex on the same day (*r* = 0.104) and computer use being correlated higher ratings of mental exhaustion (*r* = 0.165), suggesting that these are activities which crowd out most others.

Day of the week (either as a 7-day categorical variable or coded as weekend/weekday) accounted for little variance in either sleep parameters or daily experiences. School attendance, work, public transportation use, and ratings of days as mentally exhausting had the largest weekly variability, in line with social conventions scheduling free time for the weekend. Still, late sleep and interesting or pleasurable experiences tended to occur on either Friday or Saturday while work and chores were most frequently reported during the middle of the week.

### Within-participant effects

Within-participant coefficients reflect the difference in sleep characteristics within the same person after having had a certain daily experience, in line with a causal interpretation (see Methods for limitations).

The sleep characteristic most frequently affected by daily experiences was sleep onset timing, which tended to be later after pleasurable or interesting activities.

Significantly (after multiple comparisons) later sleep onset was observed after spending time in the company of others (unstandardized regression coefficient *B* = 0.302 hours, *P* = .002), alcohol consumption (*B* = 0.841 hours, *P* < .001), and days which were self-rated as more interesting (*B* = 0.118 hours per Likert point, *P* < .001), happier (*B* = 0.089 hours per Likert point, *P* < .001), or more physically exhausting (*B* = 0.05 hours per Likert point, *P* = .002). Later sleep onset after sex (*B* = 0.339 hours, *P* = .005) was nominally significant but did not pass the stringent correction for multiple comparisons. Conversely, significantly earlier sleep onset was observed after desktop computer use (*B* = −0.377 hours, *P* < .001).

Beyond changes in sleep timing 4 other significant alterations in sleep structure were observed. Spending time in the company of others was followed by increased REM latency (*B* = 8.56 minutes, *P* < .001), and alcohol consumption was followed by significantly reduced sleep latency (*B* = −0.06 log units, *P* = .005). Time at work was followed by increased TST (*B* = 23.03 minutes, *P* = .002) and N3 duration (*B* = 5.976 minutes, *P* = .003). These effects all survived correction for multiple comparisons. A fifth effect was increased REM latency (*B* = 9.739 minutes, *P* = .008) after self-reported conflicts during the day, which, however, did not survive correction for multiple comparisons. (All *P*-values presented, unless specifically indicated otherwise, remain significant after FDR correction and are presented in their uncorrected form.)

**Figure 1:**
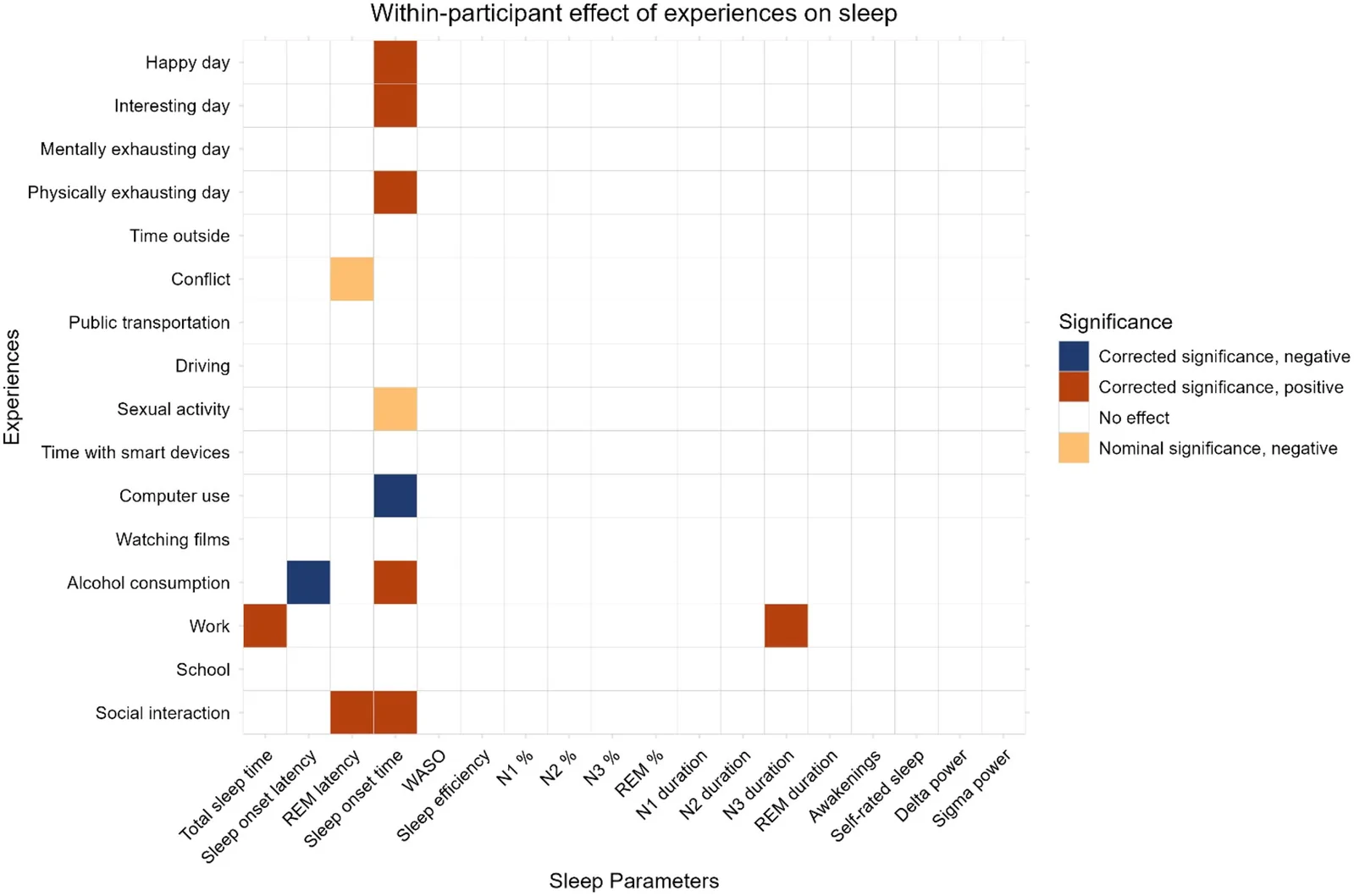
Within-participant associations between daily experiences and sleep parameters. These effects indicate that the same person’s sleep parameters are influenced by having experienced these events during the previous day. The figure illustrates results from mixed-effects models investigating the effects of daily experiences (vertical axis) on sleep parameters (horizontal axis). Dark colors show associations which survive correcting for multiple comparisons. All models are corrected for age, sex, day of the week (weekend/weekday), lagged outcomes and, with the exception of sleep timing, for the duration of previous wakefulness.

Figure 1 presents an overview of within-participant effects.

### Between-participant effects

Between-participant coefficients reflect correlations between typical sleep characteristics and the overall frequency of daily experiences or mean daily ratings. These effects do not necessarily imply causality.

Only 2 between-participant effects were observed, and none passed a formal correction for multiple comparisons. In participants with more days at work lower mean N2 duration was observed (*B* = −23.846 minutes, *P* = .003), and in participants spending more time outside mean N1 percentage was lower (*B* = −0.005 per hour outside, *P* = .008).

**Figure 2:**
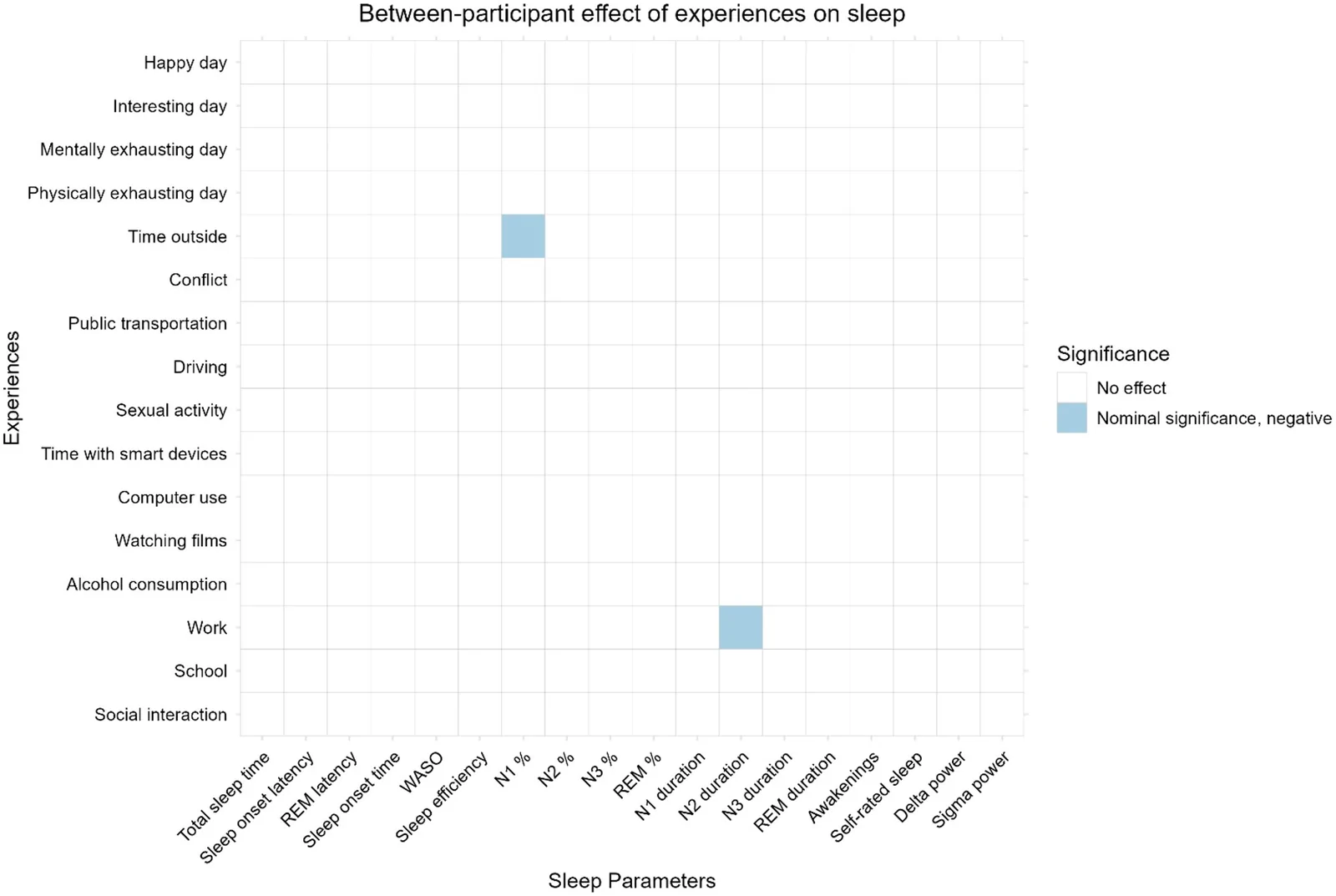
Between-participant associations between daily experiences and sleep parameters. These effects indicate associations between habitual sleep characteristics and the frequency of experiencing certain events. The figure illustrates results from mixed-effects models investigating the effects of daily experiences (vertical axis) on sleep parameters (horizontal axis). No effects are significant after correcting for multiple comparisons. All models are corrected for age, sex, day of the week (weekend/weekday), lagged outcomes and, except for sleep timing, for the duration of previous wakefulness.

Figure 2 presents an overview of between-participant effects.

All between- and within-participant effects are available in table form in the Supplementary data (item “Model output, control for homeostasis.xls”).

### Associations uncorrected for sleep homeostasis

Our models were corrected for sleep pressure, indexed by the duration of wakefulness prior to sleep. We chose this model specification because we have shown that even in the naturalistic setting of BSETS, sleep propensity is increased after extended wakefulness.^19^ Because longer wakefulness was observed after a large number of daily experiences, especially pleasant ones (Supplementary data, item Zero-order correlations.xlsx), deeper sleep after these would be expected due to homeostatic effects alone, rendering a statistical control for these necessary.

To show the importance of controlling for homeostatic effects, we re-ran our analyses without the wakefulness duration covariate. While still no significant between-participant effects were seen, more interesting days now were also followed by increased sleep propensity (reduced SOL, WASO, awakenings, N1 and N2 duration and increases in SE and delta power), mentally exhausting days were followed by increased N3 sleep (Figures S1 and S2, the full model outputs are available in the Supplementary data, item “Model output, no control for homeostasis.xls”). These effects could naively be interpreted as the experiences themselves leading to increased sleep propensity. However, their correlation with wakefulness duration (Supplementary data, item “Zero-order correlations.xlsx”) and the disappearance of their effects after controlling for the latter indicates that they merely index homeostatic effects and evidence for any sleep-enhancing effects of the experiences themselves remains scarce.

### Idiographic effects

An idiographic effect, operationalized here as significantly better fit using a random slopes compared to a random intercepts model, indicates that the effect of daily experiences on sleep varies across individuals. Evidence for such effects was found in the case of all effects on sleep timing, except for social interaction, but not for the effects on other sleep variables.

Next, we calculated correlations between individual slopes and traits to identify the individual characteristics which modify the effect of experiences on sleep. These were always found to be related to sleep habits, specifically chronotype (assessed by free day midsleep corrected for oversleeping [MSFsc] in the Munich Chronotype Questionnaire,^43^ a 19-question scale examining the timing of habitual sleep and wakefulness), napping and co-sleeping. A later chronotype predicted a greater effect of a happy day (*r* = 0.39) but a smaller effect of a physically exhausting day (*r* = −0.31) and computer use (*r* = −0.23) on sleep timing. The ­effect of alcohol consumption on sleep timing was greater in those who napped more frequently (*r* = 0.2) or longer (*r* = 0.2) but smaller in those more frequently co-sleeping with others (*r* = −0.18). Longer napping also predicted a smaller effect of computer use on sleep onset timing (*r* = −0.21). While the random slopes model also fit better for the effects of interesting days on sleep timing, we did not find traits which correlated significantly with individual effects.

## Discussion

In this study, we used a time-lagged within-participant design to investigate the effect of a large array of daily experiences on objectively and subjectively measured metrics of sleep. Our main finding is that daily experiences principally affect the timing rather than structure of sleep. After a wide array of pleasurable experiences such as social activity and a day generally rated as more interesting, happy or physically exhausting, sleep tended to occur later. Although pleasurable experiences are most likely to occur on the weekend (Table 1), these effects are significant even if day of the week is controlled for. A possible explanation for this finding is that pleasurable experiences are timed for the evening hours and function as zeitgebers to extend wakefulness. In addition, the emotional processes inherent to pleasurable experiences could serve as arousal inputs and override both the circadian and the homeostatic mechanisms involved in sleep initiation.^44^

Three main categories of daily experiences had an effect on sleep structure which survived stringent statistical control. The first such effect was related to alcohol consumption. Ethanol, the active compound of alcoholic beverages, has a GABAmimetic profile with a prominent depressing effect on central nervous system activity and complex interaction with ligand-gated ion channels,^45^^,^^46^ and was shown to cause decreased SOL and increased sleep fragmentation in experimental studies.^23^ In our study, we replicated findings about decreased SOL, but found no effect on sleep fragmentation. While precise dosage was not recorded, an analysis of the free-form evening diaries suggests that most alcohol consumption in BSETS occurred in social settings in relatively modest quantities. Low dosage can explain the mild hypnotic effect of alcohol consumption with no detriment to overall sleep quality.

Second, we also found significantly longer TST and NREM3 duration after work, but other than this no effect of school attendance, computer use, or self-reported mental exhaustion on sleep structure. This finding coheres with the broader literature, recently reviewed by Cerasuolo et al.^22^ which found mixed evidence at best (visually summarized on Figure S3) for the effects of mental strain on sleep. Workplace attendance was arguably the most challenging and least self-selected and self-paced experience ­related to cognitive demand in BSETS, and its association with increased sleep duration suggests that in a sufficiently high dosage mental exhaustion may have some effect on sleep duration and intensity.

Third, we found increased REM latency after social activity, an isolated finding which, however, remained significant after corrections for multiple testing. Self-reported conflicts during the day had a similar effect, albeit not significant after controlling for multiple comparisons (*P* = .008). Intraindividual (day-to-day) variability in the latency of REM sleep and its causes have been rarely studied in the literature. Whereas recent evidence suggests that physical activity might increase REM latency,^47^ here we reveal a similar effect of social activity and possibly conflicts. Available evidence only allows a highly speculative interpretation of this finding, which could be based on the social bonding theory of REM sleep,^48^ suggesting that the urge for REM sleep is reduced after social interactions. Given the recent increase in the interest in the social aspects of dreaming,^49^ our findings on the relationship between social interactions and delayed emergence of REM sleep during the subsequent night might fuel the renewed interest in REM latency within the context of behavioral regulatory processes.

While we found a significant effect of these 3 categories of daily experiences on sleep structure, several effects reported in the previous literature were not replicated in our study. For example, in contrast to pioneering studies like those of Horne and Minard^20^ we found no evidence that enhanced wakefulness (based on participants’ self-reports) affected the structure of subsequent sleep. While sleep occurred later after days rated as happier or more interesting, no effect on sleep structure was significant after controlling for homeostatic effects. Additionally, despite widespread belief that sexual intercourse causes somnolence, especially in men,^50–52^ and the penetration of this idea into popular media (https://tvtropes.org/pmwiki/pmwiki.php/Main/PostCoitalCollapse), we found no effect of sexual intercourse on sleep structure, including no evidence for reduced SOL. Diary-based studies^27^ of this phenomenon may be biased by recall effects and prior beliefs if participants believe that intercourse causes somnolence and self-report SOL and sexual activity at the same time. After a small early study,^53^ ours is the second to investigate these effects with objective methods, and, in contrast to self-reports, found no effect.

Besides their direct effects on sleep structure, daily experiences can also have an indirect effect through extending wakefulness and consequently inducing a homeostatic increase in sleep propensity.^37^ Naïve models not accounting for homeostatic effects (Figures S1 and S2) indeed found substantial evidence in favor of the sleep-promoting effects of experiences. These models, however, do not separate the direct effects of experiences from the indirect homeostatic effects they cause by extending wakefulness. Therefore, in our main models, we used wakefulness duration as a covariate to eliminate indirect homeostatic effects. In these models using appropriate controls, almost all apparent effects of daily experiences on the structure of subsequent sleep disappeared, illustrating the importance of isolating homeostatic effects on sleep and the possibility of confounding even in time-lagged, within-participant analyses.^36^

We found evidence that the effects of daily experiences on sleep timing are idiographic, that is, they vary from individual to individual. Sleep habits emerged as the most important factors that affected how strongly daily experiences impact subsequent sleep. Lower sleep pressure in the evening (due to late chronotype or more frequent napping) generally increased the sleep-delaying effects of pleasurable experiences (happy days, alcohol consumption), with an opposite effect on strenuous experiences (physically exhausting day, computer use). Co-sleeping reduced the sleep-delaying effect of alcohol consumption, in line with previous evidence about co-sleeping being associated with more regular sleep schedules.^54^ These findings suggest that the zeitgeber ­effect of pleasurable experiences is modified by homeostatic effects and other zeitgebers. If there is less sleep pressure in the evening (due to chronotype or napping effects), there is more room for extending sleep after pleasurable experiences and less need for immediate sleep when recent experiences would otherwise call for it. The need to synchronize sleep with a bed partner, however, has an opposite effect.

Our study has a number of limitations. The most important limitation concerns the way daily experiences were recorded in BSETS. Unlike in an experimental study, which administers a carefully planned intervention even if its ecological validity is questionable, BSETS relied on participants self-reporting daily experiences as they saw them. While this intentional design feature increases ecological validity, it also means that the dosage of daily experiences in BSETS is hard to quantify, their timing is not explicitly recorded and usually not recoverable from daily diaries. This is especially limiting for alcohol consumption, for which the dosage and timing was not precisely recorded. A further limitation is that a full sampling of all possible daily experiences is not possible: while some experiences with hypothesized sleep-modifying effects were recorded (eg, sex or mental strain), others were not (such as stress). Finally, our findings investigate the effects of typically moderate, often self-paced enhancements or fluctuations in the intensity of wakeful experiences on sleep. They do not rule out that very intensive enhancements of wakefulness promote sleep; however, these are less feasible as the basis of regularly administered nonpharmacological interventions.

Our findings have potential clinical implications. One goal of the current study was to test if it is feasible to design minimally invasive interventions to improve sleep which prescribe behavioral modifications within the normally observable range of participants (such as an increase in time spent outside to the weekly maximum or a reduction in time spent with smart devices to the weekly minimum). Although nominally a volunteer sample, a substantial proportion of BSETS participants had clinically significant levels of insomnia complaints, in line with previous evidence^6^ about the presence of such symptoms in the general population. While we found strong evidence that the timing of sleep is affected by daily experiences, less evidence was found that the structure or quality of sleep is improved after specific daily experiences. This observation calls for realism about the possible sleep-promoting effects of simple nonpharmacological interventions consisting of limiting certain common daily experiences (such as work or smart device use) or increasing others (such as time spent outside, enriching time spent awake, or spending more time in the company of others). While nonpharmacological interventions can be effective in promoting sleep, either they need to rely on well-supported homeostatic effects by extending the duration of wakefulness, or they need to be more intensive than simply fine-tuning the normal daily routine of those with sleep problems.

## Supplementary Material

kaag003_Supplementary_Data

## Data Availability

Raw data to replicate our analyses are available at https://osf.io/92agb/.

## Transparency statements

1. Study registration. This study was not formally registered.
2. Analytic plan pre-registration. The analysis plan was not formally pre-registered.
3. Analytic code availability. Analytic code used to conduct the analyses presented in this study are available in a public archive: https://osf.io/92agb/
4. Materials availability. All materials used to conduct the a study are available in a public archive: https://osf.io/92agb/
