# Supplementary material for: Daily experiences affect the timing rather than the structure of sleep
Source: Ann Behav Med. 2026 Feb 19;60(1):kaag003. doi: 10.1093/abm/kaag003 (PMC13017393; doi:10.1093/abm/kaag003)
Supplement: kaag003_Supplementary_Data [file kaag003_supplementary_data.docx]

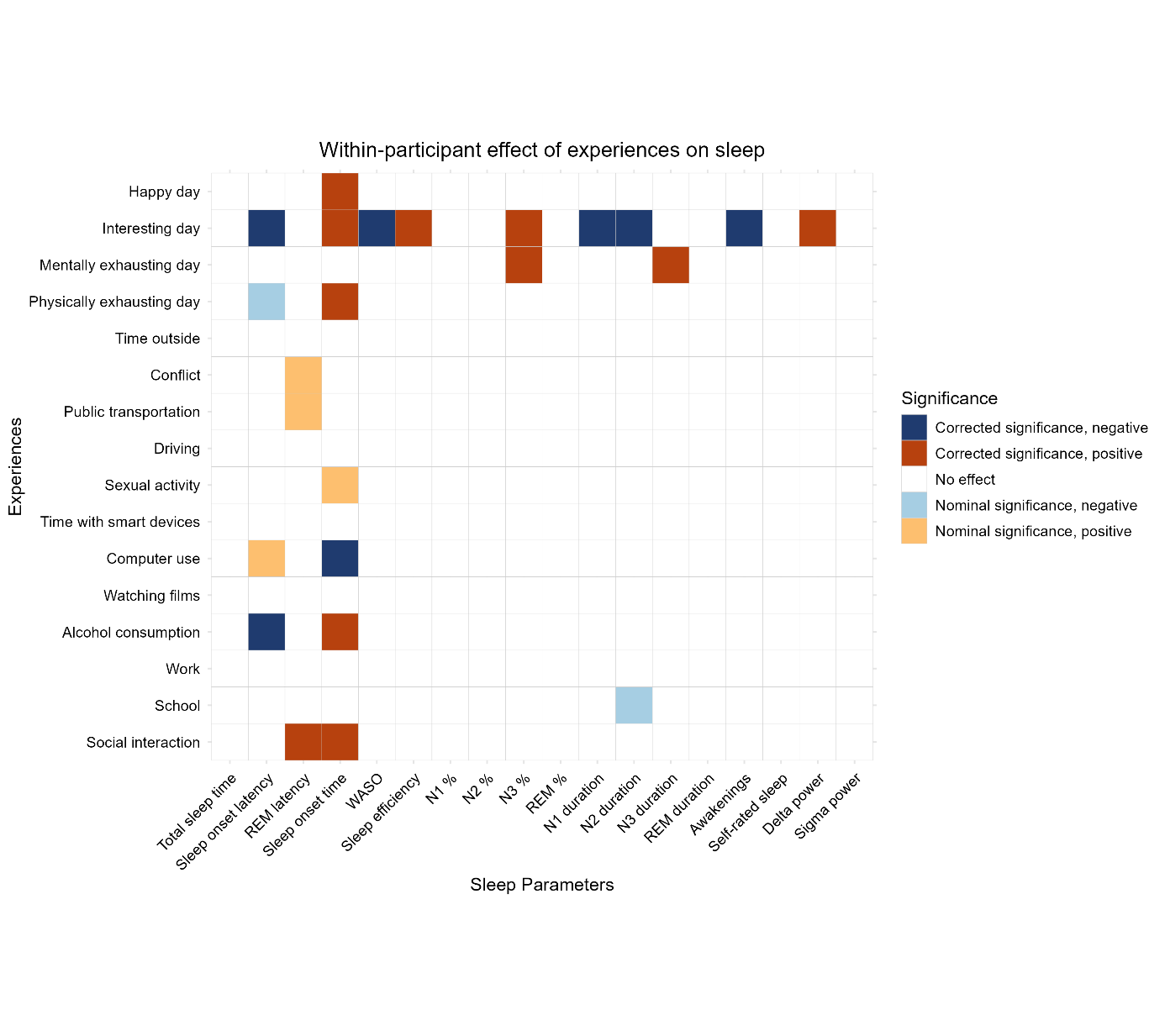


**Supplementary figure S1**. Within-participant associations between daily experiences and sleep parameters. These effects indicate that the same person’s sleep parameters are influenced by having experienced these events during the previous day. The figure illustrates results from mixed-effects models investigating the effects of daily experiences (vertical axis) on sleep parameters (horizontal axis). Dark colors show associations which survive correcting for multiple comparisons. All models are corrected for age, sex and day of the week (weekend/weekday) and lagged outcomes but, unlike those reported in the main manuscript, not for the duration of previous wakefulness.


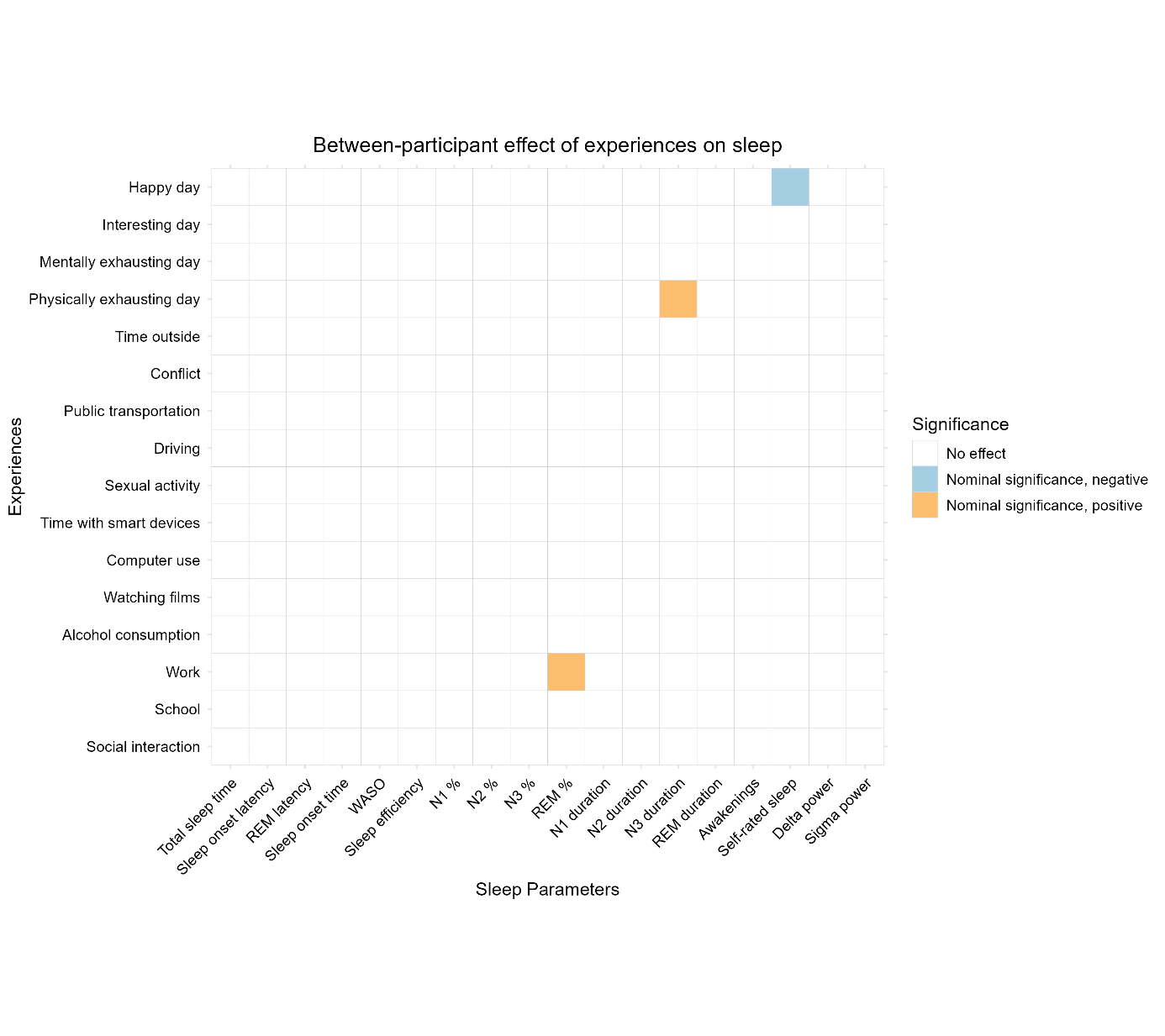


**Supplementary figure S2**. Between-participant associations between daily experiences and sleep parameters. These effects indicate associations between habitual sleep characteristics and the frequency of experiencing certain events. The figure illustrates results from mixed-effects models investigating the effects of daily experiences (vertical axis) on sleep parameters (horizontal axis). No effects are significant after correcting for multiple comparisons. All models are corrected for age, sex and day of the week (weekend/weekday) and lagged outcomes, but, unlike those reported in the main manuscript, not for the duration of previous wakefulness.


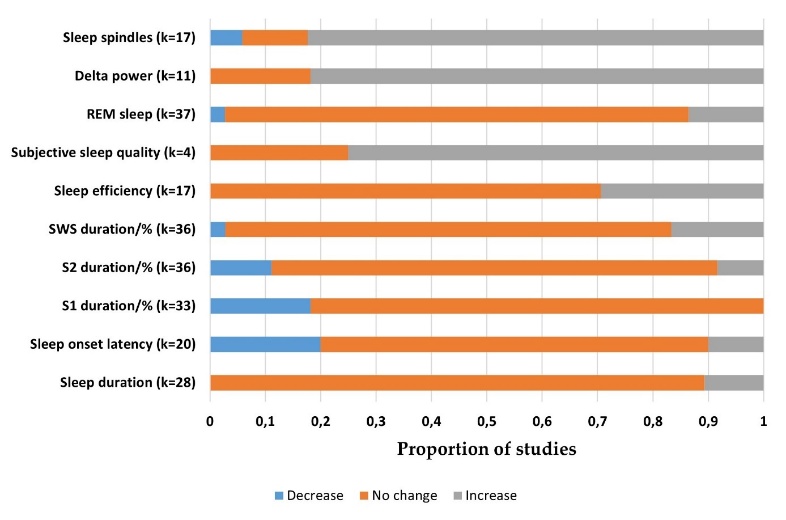


**Supplementary figure S3.** Effects of intensive cognitive activity on sleep characteristics, based on the systematic review by Cerasuolo et al [[26]](https://sciwheel.com/work/citation?ids=14100474&pre=&suf=&sa=0&dbf=0). Stacked bars show the proportion of studies reporting decreases, no change, or increases in sleep characteristics following cognitive activity. The chart shows a selection of sleep characteristics based on the size of the available literature in the original study and compatibility with BSETS results. The number of available studies is indicated in parentheses.
